# Spatial intratumoural heterogeneity in the expression of GIT1 is associated with poor prognostic outcome in oestrogen receptor positive breast cancer patients with synchronous lymph node metastases

**DOI:** 10.12688/f1000research.12393.2

**Published:** 2018-02-14

**Authors:** Ibai Goicoechea, Ricardo Rezola, María Arestin, María M. Caffarel, Ana Rosa Cortazar, Lorea Manterola, Marta Fernandez-Mercado, María Armesto, Carla Sole, Erika Larrea, Angela M. Araujo, Nerea Ancizar, Arrate Plazaola, Ander Urruticoechea, Arkaitz Carracedo, Irune Ruiz, Isabel Alvarez Lopez, Charles H. Lawrie

**Affiliations:** 1 Molecular Oncology Group, Biodonostia Research Institute, San Sebastián, 20014, Spain; 2 Department of Pathology and Anatomy, Onkologikoa- Instituto Oncológico, San Sebastián, 20014, Spain; 3 IKERBASQUE, Basque Foundation for Science, Bilbao, 48013, Spain; 4 CIC bioGUNE, Derio, 48160, Spain; 5 Oncology Department, University Hospital Donostia, San Sebastián, 20014, Spain; 6 Onkologikoa- Instituto Oncológico, San Sebastián, 20014, Spain; 7 Department of Biochemistry and Molecular Biology, University of the Basque Country, Leioa , 48940, Spain; 8 Department of Pathology and Anatomy, University Hospital Donostia, San Sebastián, 20014, Spain; 9 Radcliffe Department of Medicine, University of Oxford, Oxford, OX3 9DU, UK

**Keywords:** GIT1, breast cancer, immunohistochemistry, biomarker, lymph node

## Abstract

**Background**: The outcome for oestrogen receptor positive (ER+) breast cancer patients has improved greatly in recent years largely due to targeted therapy. However, the presence of involved multiple synchronous lymph nodes remains associated with a poor outcome. Consequently, these patients would benefit from the identification of new prognostic biomarkers and therapeutic targets. The expression of G-protein-coupled receptor kinase-interacting protein 1 (GIT1) has recently been shown to be an indicator of advanced stage breast cancer. Therefore, we investigated its expression and prognostic value of GIT1 in a cohort of 140 ER+ breast cancer with synchronous lymph node involvement.

**Methods**: Immunohistochemistry was employed to assess GIT1 expression in a tissue microarray (TMA) containing duplicate non-adjacent cores with matched primary tumour and lymph node tissue (n=140). GIT1 expression in tumour cells was scored and statistical correlation analyses were carried out.

**Results**: The results revealed a sub-group of patients that displayed discordant expression of GIT1 between the primary tumour and the lymph nodes (i.e. spatial intratumoural heterogeneity). We observed that loss of GIT1 expression in the tumour cells of the metastasis was associated with a shorter time to recurrence, poorer overall survival, and a shorter median survival time. Moreover, multivariate analysis demonstrated that GIT1 expression was an independent prognostic indicator.

**Conclusions**: GIT1 expression enabled the identification of a sub-class of ER+ patients with lymph node metastasis that have a particularly poor prognostic outcome. We propose that this biomarker could be used to further stratify ER+ breast cancer patients with synchronous lymph node involvement and therefore facilitate adjuvant therapy decision making.

## Introduction

Breast cancer is the most common type of cancer among Western women, and every year around 450,000 new cases are diagnosed in Europe alone ^1^. Breast cancer is recognised as a very heterogeneous disease and several different subgroups have been identified on the basis of hormone receptor and Her2 status, or more recently by gene expression profiling ^2^. Each subgroup shows different pathological, clinical and molecular characteristics, which in turn have different therapeutic options and prognostic outcome. The oestrogen-positive (ER+) breast cancer subtype is the most common of these subtypes representing around 70% of all cases. Although the majority of ER+ cases respond to anti-oestrogen treatments, some are resistant to endocrine therapies, in particular those patients with involved lymph nodes and distant metastases at time of presentation ^3, 4^. Indeed, the presence of synchronous lymph node metastasis is a strong indicator of poor prognostic outcome ^5^. Therefore, there is a clear clinical need to identify new therapeutic targets and biomarkers for this group of patients including those that do not relapse.

It has been shown recently that advanced stage breast cancer and involved lymph nodes express high mRNA levels of G-protein-coupled receptor kinase-interacting protein 1 (GIT1) ^6^, however, this study did not examine GIT1 protein expression, with the exception of nine tumours. As immunohistochemistry (IHC) exhibits greater potential for clinical application we decided to evaluate GIT1 immunoreactivity in an extensive cohort of ER+ breast cancer cases with synchronous lymph node (LN) involvement (n=140). We evaluated the association of GIT1 expression in the primary tumour and in the synchronous LN with clinical features and disease aggressiveness.

## Methods

### Patient cohort

Primary site and lymph node metastasis tissue samples were obtained from the Pathology Department of Donostia University Hospital from 140 breast patients who were diagnosed between 2000 and 2006, with follow-up data until 2014 with a median follow-up of 8 years and 8 months. Written consent was obtained from patients for the inclusion of their samples in this study and samples were collected in accordance with the Declaration of Helsinki, and approved by local ethics committees (Comite Etico de Investigación Clínica de Euskadi (CEIC-E)). Cases were selected and re-reviewed by two experienced breast pathologists independently. Clinical data was only available for 104 of these patients ( Dataset 1). All patients were women with an age range between 27 and 86 years old (median age 58 years old). All primary site tumours were ER+ and 91% of them PR+. Eighty-six percent of patients presented with invasive carcinoma of no special type (NST) and 11% with invasive lobular carcinoma (ILC). Histological grades varied from grade I (21%), grade II (55%) to grade III (15%), according to the Elston-Ellis modification of Scarff-Bloom-Richardson grading system. All patients were surgically treated either by tumorectomy or by mastectomy with axillary lymphadenectomy. All patients underwent hormone therapy with the majority of them receiving radiotherapy (90%) and adjuvant chemotherapy (75%). Table 1 summarizes the clinical and histological characteristics of patients as we previously described ^7^.

**Table 1.** Clinical and histological characteristics of patients used in study.

Features			n (%)
Histological subgroup	NSC		90 (86.5)
	ILC		12 (11.5)
	other		2 (1.9)
Tumour size (mm)	<20		40 (38.8)
	20–40		53 (51.5)
	>40		10 (9.7)
Number of affected lymph nodes	<5		70 (68.0)
	5–10		24 (23.3)
	>10		9 (8.7)
Histological grade	I		22 (21.4)
	II		57 (55.3)
	III		15 (14.6)
	Unknown		9 (8.7)
Vascular lymphatic infiltration	No		81 (78.6)
	Yes		22 (21.4)
Immunohistochemical initial status	ER	Positive	103 (100.0)
		Negative	0 (0.0)
	PR	Positive	94 (91.3)
		Negative	9 (8.7)
	HER2	Score 0	46 (44.7)
		Score 1	43 (41.7)
		Score 2	7 (6.8)
		Score 3	7 (6.8)
Treatment	Surgery	Tumorectomy	55 (53.4)
		Mastectomy	49 (47.6)
	Radiotherapy	No	8 (7.8)
		Breast conserving therapy	55 (53.4)
		Thoracic wall	39 (37.9)
	Adjuvant chemotherapy	NO	25 (24.3)
		YES	78 (75.7)
	Hormone therapy	NO	1 (1.0)
		Tamoxifen	34 (33.0)
		AI	25 (24.3)
		Tmx -> AI	42 (40.8)
Follow up	Recurrence	NO	77 (74.8)
		YES	27 (26.2)
	Distant metastasis	NO	73 (70.9)
		YES	31 (30.1)
	Death	NO	69 (67.0)
		YES	36 (35.0)

[i] Abbreviations: n (%) = number of patients (percentage within each feature), AI = Aromatase inhibitors, Tmx -> AI = Tamoxifen followed by Aromatase inhibitors. NST, invasive carcinoma of no special type; ILC, invasive lobular carcinoma. p values calculated with Chi-square contingency test.

### Tissue microarray construction and immunohistochemistry (IHC)

Representative areas of high tumour load (>70%) were selected after H&E staining and two 1.5mm punch biopsies taken from both primary tumour and lymph node and arrayed non-adjacently to reduce staining bias using a Manual Tissue Arrayer MTA-1 (Beecher Instruments, USA).

Immunohistochemical (IHC) staining was carried out on 5µm slices manually using the Immunohistochemistry Accessory Kit of Bethyl Laboratories (Montgomery, USA). Slides were deparaffinized in xylene and blocked in peroxidase for 30 min. Antigen retrieval was carried out in Epitope Retrieval Buffer (Bethyl Laboratories, Montgomery, USA). Slides were blocked in BSA for 30 min and then incubated for 1h at room temperature with anti-GIT1 rabbit polyclonal antibody (1:100) (IHC-00527 (lot #001), Bethyl Laboratories, Montgomery, USA). Even though this is the same antibody used by the study of Chan *et al.* ^6^, and raised against a GIT1-specific peptide immunogen, we cannot rule out cross-reactivity against other non-GIT1 epitopes. After 1h incubation with secondary anti-Rabbit IHC Antibody and DAB substrate. The slides counterstained with hematoxylin.

**Figure 1.**
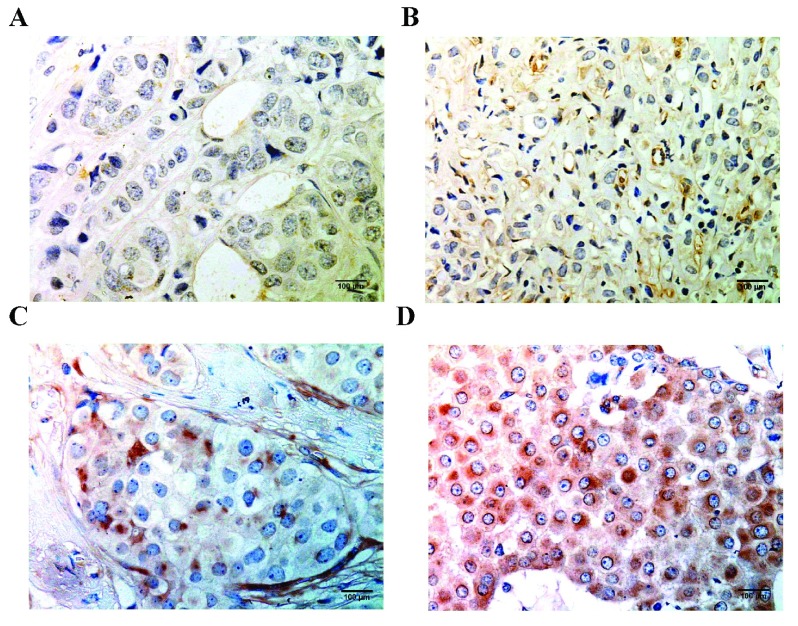
Example of GIT1 expression patterns found in ER+ breast cancer. Images were enhanced from the original ( Supplementary Figure S2). Representative intensity staining of GIT1 expression (primary tumour) depicting **A**. negative (score=0); **B**. weak (score=1); **C**. moderate (score=2); and **D**. strong (score=3). Magnification ×400.

GIT1 expression was scored in a blinded fashion by an experienced breast pathologist according to tumour cell staining intensity and categorical scores assigned as follows; 0= negative (0%); 1=1–10%; 2= 11–50%; 3= >50% ( Dataset 2). Scores between non-adjacent cores were combined and categorised according to following criteria; GIT1 negative (combined score <1); moderate GIT1 expression (combined score 1–2); and high GIT1 expression (score >2). Examples of the different staining categories are shown in Figure 1 and Supplementary Figure S2. A selection of cases (n=8) were examined both as whole biopsy sections and TMAs, all scoring was concordant. Examples of whole section IHC staining with GIT1 are shown in Supplementary Figure S1 and Supplementary Figure S3.

Clinical data of patient cohort Table shows patients (numbered from 1 to 105) and their clinical features including histological subgroup, tumour size, number of affected lymph nodes, histological grade, vascular lymphatic infiltration, immunohistochemical initial status, treatment and patient follow up. Click here for additional data file. Copyright: © 2018 Goicoechea I et al. 2018 Data associated with the article are available under the terms of the Creative Commons Zero "No rights reserved" data waiver (CC0 1.0 Public domain dedication).

GIT1 scoring Table shows patients (numbered from 1 to 142) and associated primary tumour and lymph node GIT1 scoring. Categorical scores are assigned as follows according to tumour cell staining intensity; 0= negative (0%); 1=1–10%; 2= 11–50%; 3= >50%. Click here for additional data file. Copyright: © 2018 Goicoechea I et al. 2018 Data associated with the article are available under the terms of the Creative Commons Zero "No rights reserved" data waiver (CC0 1.0 Public domain dedication).

### Public dataset analysis

GIT1 gene expression levels were analyzed using publicly available databases. For this analyses we interrogated the TCGA dataset which included 525 mixed breast cancer tumours and 22 normal breast samples ( Dataset 3) ^8^, 2000 mixed breast tumours ( Dataset 4) ^9^, 570 metastatic breast tumours ( Dataset 5) ^10^, 252 lymph-node negative breast cancer patients ( Dataset 6) ^11^, 67 triple negative breast cancer patients ( Dataset 7) ^12^, and 19 primary breast cancer and 19 brain metastasis from HER2 positive breast cancer patients ( Dataset 8) ^13^.

Series mRNA expression matrix and clinical data information GIT1 Expression Dataset consisting of 522 primary tumors, 3 metastatic tumors, and 22 tumor-adjacent normal samples. Data was median centered by genes. Platform: Affymetrix Human Genome U133A Array. Publicly available from https://tcga-data.nci.nih.gov/docs/publications/brca_2012/. Click here for additional data file. Copyright: © 2018 Goicoechea I et al. 2018 Data associated with the article are available under the terms of the Creative Commons Zero "No rights reserved" data waiver (CC0 1.0 Public domain dedication).

Series mRNA expression matrix Expression Dataset consisting of 2000 breast carcinoma. Platform: Affymetrix Human HT-12 V3 Array. Publicly available from http://www.cbioportal.org/study?id=brca_metabric#summary Click here for additional data file. Copyright: © 2018 Goicoechea I et al. 2018 Data associated with the article are available under the terms of the Creative Commons Zero "No rights reserved" data waiver (CC0 1.0 Public domain dedication).

Series mRNA expression matrix Expression Dataset consisting of one hundred fifty-four (154) invasive breast carcinoma samples and 4 normal breast samples. Platform: Agilent UNC Perou Lab Homo sapiens 1X44K Custom Array. Publicly available from Gluck Breast dataset ( https://www.oncomine.org) Click here for additional data file. Copyright: © 2018 Goicoechea I et al. 2018 Data associated with the article are available under the terms of the Creative Commons Zero "No rights reserved" data waiver (CC0 1.0 Public domain dedication).

Series mRNA expression matrix Expression Dataset consisting of 252 lymph-node negative breast cancer samples. Platform: Affymetrix Human Genome U133A Array. Publicly available from https://www.ncbi.nlm.nih.gov/geo/query/acc.cgi?acc=gse2034
Copyright: © 2018 Goicoechea I et al. 2018 Data associated with the article are available under the terms of the Creative Commons Zero "No rights reserved" data waiver (CC0 1.0 Public domain dedication).

Series mRNA expression matrix Expression Dataset consisting of 67 triple negative breast cancer samples. Platform: Affymetrix Human Genome U133A Array. Publicly available from https://www.ncbi.nlm.nih.gov/geo/query/acc.cgi?acc=GSE31519
Copyright: © 2018 Goicoechea I et al. 2018 Data associated with the article are available under the terms of the Creative Commons Zero "No rights reserved" data waiver (CC0 1.0 Public domain dedication).

Series mRNA expression matrix Expression Dataset consisting of 19 HER2+ brain metastasis breast cancer samples and 19 HER2+ non-metastatic breast cancer samples. Platform: Affymetrix Human X3P Array. Publicly available from https://www.ncbi.nlm.nih.gov/geo/query/acc.cgi?acc=GSE43837
Copyright: © 2018 Goicoechea I et al. 2018 Data associated with the article are available under the terms of the Creative Commons Zero "No rights reserved" data waiver (CC0 1.0 Public domain dedication).

### Statistical analysis

**Figure 2.**
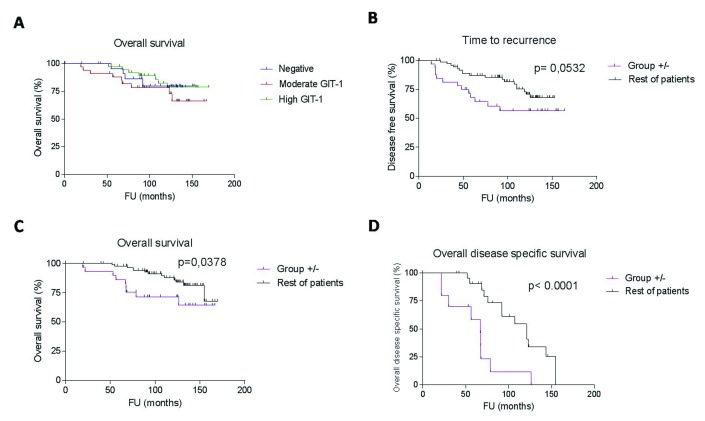
Kaplan-Meier survival curves of ER+/LN+ breast cancer cases according to GIT1 expression. Curves were compared by univariate (log-rank) analysis. **A**. Cases sub-classified according to expression levels of GIT1 in the primary tumour (or LN) were not significantly different. Cases that were GIT1 +/- (n=31) had a shorter time to recurrence ( **B**) and overall survival ( **C**), and disease specific survival ( **D**) than non GIT1+/- cases (n=73).

**Figure 3.**
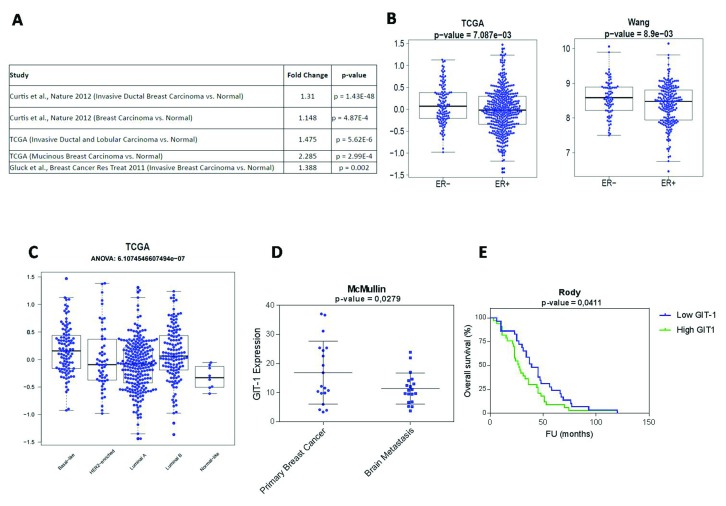
GIT1 expression in different breast cancer subtypes. **A**. GIT1 expression is significantly higher in breast cancer samples compared to normal breast cancer tissue. *In silico* meta-analysis of five databases representing 3452 cases. **B**. In two independent datasets GIT1 expression was lower in ER positive compared to ER negative tumours. **C**. GIT1 expression is highest in the basal breast cancer subtype which represents ER-cases (ANOVA analysis). **D.** GIT1 expression is lower in brain metastasis than primary breast tumour sites. **E**. Survival analysis of triple negative breast cancer patients show low GIT1 expression is associated with poorer clinical outcome. Patients were sub-classified on the basis of median GIT1 expression levels (univariate log rank test). Unless otherwise specified analysis was carried out by independent *t*-test.

Chi-square statistical test was used to determine association between GIT1 expression and lymph node metastasis ( Table 3). Fisher’s exact test and Chi-square test were used to associate GIT1 expression with clinicopathological features ( Table 1, Table 2 and Table 4). For survival analysis, Kaplan-Meier curves and univariate Log-rank (Mantel-Cox) analysis were performed ( Figure 2 and Figure 3E). Statistical analyses were performed using GraphPad Prism v5.03 (GraphPad Software, La Jolla, CA, United States). Cox regression for multivariate analysis was performed with SPSS Statistics 20 (IBM, New York, USA) ( Table 5).

For public dataset analysis, expression data were analyzed by t-test when comparing 2 groups or Anova when comparing more than 2 groups ( Figure 3). Data was analyzed using GraphPad Prism v5.03 (CA, United States) and R (R Foundation for Statistical Computing, Vienna, Austria).

## Results

### Analysis of GIT1 expression in primary tumours

We observed both cytoplasmic and membrane expression of GIT1 in tumour cells of varying intensities, along with some perinuclear localisation and some weaker signal in stromal cells ( Figure 1). This staining pattern is consistent with that previously observed ^6, 14^.

In order to ascertain the association of tumoural GIT1 expression with clinicopathological characteristics, we scored the expression according to intensity and % positive tumour cells in each case (based on two cores) as negative, moderate and high ( Figure 1). Out of the 140 primary tumour specimens, we observed high expression of GIT1 in 47 cases (34%), moderate expression in 58 cases (42%) and no GIT1 expression in 35 cases (25%). There was no significant association between GIT1 expression and the 2012 WHO defined histological subtype (i.e. invasive carcinoma of no special type (NST; n=90 (86.5%)), invasive lobular carcinoma (ILC; n=12 (11.5%)) or other). Neither were there significant associations with hormone receptor status, tumour size, number of affected lymph nodes, histological grade or the presence of vascular lymphatic infiltrate ( Table 2). We observed no significant difference in the overall survival (OS) ( Figure 2A), or time to recurrence in patients according to the level of GIT1 expression in these primary tumours (data not shown).

**Table 2.** Association between GIT1 expression levels and clinicopathological features.

Features			GIT1 expression, n (%)			p value
			Negative	Moderate	High	
Histological subgroup	Ductal		22 (84.6)	34 (91.9)	34 (82.9)	0.6436
	Lobular		3 (11.5)	3 (8.1)	6 (14.6)	
	other		1 (3.8)	0 (0.0)	1 (2.4)	
Tumour size (mm)	<20		10 (38.5)	16 (44.4)	14 (34.1)	0.6732
	20-40		14 (53.8)	18 (50.0)	21 (51.2)	
	>40		2 (7.7)	2 (5.6)	6 (14.6)	
Number of affected lymph nodes	>5		18 (69.2)	25 (69.4)	27 (65.9)	0.823
	5-10		7 (26.9)	8 (22.2)	9 (22.0)	
	>10		1 (3.8)	3 (8.3)	5 (12.2)	
Histological grade	I		8 (30.8)	5 (13.9)	9 (22.0)	0.5075
	II		13 (50.0)	21 (58.3)	23 (56.1)	
	III		4 (15.4)	7 (19.4)	4 (9.8)	
	Unknown		1 (3.8)	3 (8.3)	5 (12.2)	
Vascular lymphatic infiltration	No		21 (80.8)	27 (75.0)	33 (80.5)	0.8035
	Yes		5 (19.2)	9 (25.0)	8 (19.5)	
Immunohistochemical initial status	ER	Positive	26 (100.0)	36 (100.0)	41 (100.0)	n.a.
		Negative	0 (0.0)	0 (0.0)	0 (0.0)	
	PR	Positive	24 (92.3)	34 (94.4)	36 (87.8)	0.5748
		Negative	2 (7.7)	2 (5.6)	5 (12.2)	
	HER2	Score 0	12 (46.2)	16 (44.4)	18 (43.9)	0.4251
		Score 1	9 (34.6)	15 (41.7)	19 (46.3)	
		Score 2	4 (15.4)	1 (2.8)	2 (4.9)	
		Score 3	1 (3.8)	4 (11.1)	2 (4.9)	
Treatment	Surgery	Tumorectomy	15 (57.7)	17 (47.2)	23 (56.1)	0.649
		Mastectomy	11 (42.3)	19 (52.8)	18 (43.9)	
	Radiotherapy	No	0 (0.0)	4 (11.1)	4 (9.8)	0.3749
		Breast conserving therapy	15 (57.7)	16 (44.4)	24 (58.5)	
		Thoracic wall	11 (42.3)	15 (41.7)	13 (31.7)	
	Adjuvant chemotherapy	NO	6 (23.1)	9 (25.0)	10 (24.4)	0.9721
		YES	20 (76.9)	26 (72.2)	31 (75.6)	
	Hormone therapy	NO	0 (0.0)	1 (2.8)	0 (0.0)	0.1941
		Tamoxifen	9 (34.6)	14 (38.9)	11 (26.8)	
		AI	9 (34.6)	9 (25.0)	7 (17.1)	
		Tmx -> AI	8 (30.8)	11 (30.6)	23 (56.1)	

[i] Abbreviations: n (%) = number of patients (percentage within each feature), AI = Aromatase inhibitors, Tmx -> AI = Tamoxifen followed by Aromatase inhibitors. p values calculated with Chi-square contingency test

### Spatial intratumoural heterogeneity of GIT1 expression in ER+ breast cancer

When we compared GIT1 expression in primary tumours with that of their counterpart lymph node metastases surprisingly we observed a significant decrease of GIT1 expression (p=0.0054, Table 3). Although both lymph node and primary biopsies showed a similar frequency of high GIT1 expression (29% vs 34% respectively), the percentage of lymph nodes with moderate GIT1 expression was lower than that of primary tumours (29% vs 41% respectively), and 43% of lymph node samples were negative for GIT1 compared with 25% of primary tumour samples ( Table 3).

**Table 3.** Relationship between GIT1 expression in primary tumour and lymph node metastasis.

	GIT1 expression, n (%)			p value
	Negative	Moderate	High	
Tumour	35 (25.0)	58 (41.4)	47 (33.6)	0.0054
Lymph node metastasis	60 (42.9)	40 (28.6)	40 (28.6)	

[i] Abbreviations: n (%) = number of patients (percentage with respect to all patients “140”). p value calculated with Chi-square contingency test.

To explore this phenomenon further, we carried out a comparative analysis of GIT1 expression between the primary tumour site and matched synchronous lymph node metastasis. We found 64 cases (63%) with concordant GIT1 expression, either both positive for GIT1 expression (scores >1, n=45 (44%), or both negative for GIT1 expression (scores<1, n=19 (19%), and 38 cases (37%) with discordant GIT1 expression between the primary tumour and the lymph node.

As intratumoural heterogeneity has been suggested to be associated with resistance to therapy and prognostic outcome in breast cancer ^15^, we investigated whether this spatial heterogeneity in GIT1 expression might be associated with clinical outcome (or clinicopathological characteristics) in our cohort. For this analysis we sub-classified cases into four groups: "group 0" cases were negative for GIT1 expression in both primary and lymph nodes (n=19); "group ++" cases were positive for GIT1 expression in both primary and lymph nodes (n=45); "group +/-" cases were positive for GIT1 expression in primary but not lymph nodes (i.e. loss of GIT1 expression; n=31); and "group -/+" cases were negative for GIT1 expression in primary but positive for GIT1 expression in lymph nodes (i.e. gain of GIT1 expression; n=7).

We did not detect any significant association between the aforementioned score and other clinicopathological features ( Table 4). We analyzed the survival of these four groups and observed that group +/- showed a tendency towards shorter time to recurrence when compared to the rest of patients (p=0.05, hazard ratio = 2.902; Figure 2B), and that these patients had a significantly poorer overall survival than the rest of the groups (p=0.03, hazard ratio = 2.996; Figure 2C). Furthermore, the disease specific survival of patients with +/- GIT1 expression was significantly worse than other patients (p<0.0001, hazard ratio = 7.423; Figure 2D) with a median survival time of only 67 months compared to 110 months, representing a reduction of ~40%. Comparing with other well defined prognostic indicators (histological grade, presence of distant metastasis) by multivariate analysis in our cohort, the loss of GIT1 expression in lymph nodes (+/- pattern) was an independent prognostic indicator (p=0.045; ( Table 5)).

**Table 4.** Comparison of GIT1 +/- patients and the rest of the patients with clinicopathological features.

Features			GIT1 expression, n (%)		p value
			+/- Group	Rest of patients	
Histological subgroup	Ductal		27 (87.1)	62 (87.3)	>0.9999
	Lobular		3 (9.7)	8 (11.3)	
	other		1 (3.2)	1 (1.4)	
Tumour size (mm)	<20		11 (35.5)	29 (40.8)	0.7416
	20–40		16 (51.6)	36 (50.7)	
	>40		4 (12.9)	6 (8.5)	
Number of affected lymph nodes	<5		23 (74.2)	46 (64.8)	0.6389
	5–10		6 (19.4)	18 (25.4)	
	>10		2 (6.5)	7 (9.9)	
Histological grade	I		5 (16.1)	16 (22.5)	0.3235
	II		17 (54.8)	40 (56.3)	
	III		7 (22.6)	8 (11.3)	
	Unknown		2 (6.5)	7 (9.9)	
Vascular lymphatic infiltration	No		22 (73.1)	58 (81.7)	0.2954
	Yes		9 (26.9)	13 (18.3)	
Immunohistochemical initial status	ER	Positive	31 (100.0)	71 (100.0)	n.a.
		Negative	0 (0.0)	0 (0.0)	
	PR	Positive	30 (96.8)	63 (88.7)	0.2701
		Negative	1 (3.2)	8 (11.3)	
	HER2	Score 0	14 (45.2)	31 (43.7)	0.2985
		Score 1	14 (45.2)	29 (40.8)	
		Score 2	0 (0.0)	7 (9.9)	
		Score 3	3 (9.7)	4 (5.6)	
Treatment	Surgery	Tumorectomy	14 (45.2)	41 (57.7)	0.2836
		Mastectomy	17 (54.8)	30 (42.3)	
	Radiotherapy	No	3 (9.7)	5 (7.0)	0.4257
		Breast conserving therapy	14 (45.2)	42 (59.2)	
		Thoracic wall	14 (45.2)	24 (33.8)	
	Adjuvant chemotherapy	NO	6 (19.4)	19 (26.8)	0.466
		YES	25 (80.6)	52 (73.2)	
	Hormone therapy	NO	0 (0.0)	1 (1.4)	0.4376
		Tamoxifen	13 (41.9)	21 (29.6)	
		AI	5 (16.1)	20 (28.2)	
		Tmx -> AI	13 (41.9)	28 (39.4)	

[i] Abbreviations: n (%) = number of patients (percentage within each feature), AI = Aromatase inhibitors, Tmx -> AI = Tamoxifen followed by Aromatase inhibitors. p values calculated with Chi-square contingency test

**Table 5.** Cox regression multivariate analysis of overall survival in patients with breast cancer.

	P value	Hazard ratio	95% CI
Distant metastasis	0.943	>10	0 - >10
Histological grade	0.127	1.811	0.84 – 3.88
Group +/-	0.045	2.759	1.02 – 7.44

[i] Table shows the relevance of presence of distant metástasis, a high histological grade (III) and being in the group +/- as predictor variables to overall survival. p values are calculated with Cox regression for multivariate analysis test

### *In silico* analysis of GIT1 expression in breast cancer

To further examine *GIT1* expression in breast cancer, we analyzed gene expression levels in several publicly available gene expression datasets. This revealed that *GIT1* expression was significantly higher in breast cancer (n=144) compared to non-tumoural tissue (n=14) ( *P*=4.87 x 10 ^-4^) ^8– 10^ ( Figure 3A), and was particularly pronounced when comparing only NST cases (n=1556) with healthy breast tissue (n=144) ( *P*=1.43 x 10 ^-48^). A comparison between the two main subtypes of breast cancer (i.e. NST and ILC) in the TCGA dataset (n=525) showed differences in expression levels with healthy breast tissue (n=64) ( *P=*5.62 x 10 ^-6^) as did the dataset of Gluck *et al* (n=154 vs 4 (breast carcinoma vs healthy control) ( *P=*0.002)) ( Figure 3A) ^10^. A comparison of *GIT1* expression between ER+ and ER-tumours in two independent Datasets demonstrated a significantly lower level of expression in ER+ tumours compared to ER- tumours. These comparisons were carried out on the TCGA dataset (n=601 (ER+) vs. n=179 (ER-) and the Wang dataset (n=209 (ER+) vs. n=77 (ER-) ^8, 11^ Figure 3B). Consistent with these findings, basal tumours, which are mostly ER negative, showed higher GIT1 expression than the rest of the molecular subgroups of breast cancer in the TCGA dataset ( *P=*6.11 x 10 ^-7^; Figure 3C).

We also looked at *GIT1* expression between the primary tumour and brain metastasis in a cohort of HER2-positive (mixed ER+ and ER- cases) breast cancers (n=19) ^13^. We observed that GIT1 expression was significantly decreased in brain metastases ( *P=*0.0279; Figure 3D). Furthermore, when we interrogated data from a publicly available cohort of triple negative breast cancer patients (n=67) ^12^, we found that patients with GIT1 expression below the median had significantly poorer prognosis regarding event-free interval than those with GIT1 levels above the median ( *P*=0.0411, hazard ratio = 1.625; Figure 3E).

## Discussion

GIT1 is a GTPase-activating protein (GAP) that act to inhibit GTPase activity of members of the ADP-ribosylation factor (Arf) family, specifically Arf1 and Arf6, by converting bound GTP to GDP ^16^. It is involved in many cellular processes including cell adhesion, migration, lamellipodia formation, cell growth and angiogenesis ^17– 20^.

In addition, GIT1 can activate many signalling pathways involved in carcinogenesis such as ERK1/2, Rho, AARF or P21-activated kinase (PAK) ^17, 21^. GIT1 has been demonstrated to be over-expressed in several cancers including hepatocellular carcinoma, colon cancer, lung cancer and melanoma ^17, 22– 25^.

In the current study, we ascertained the clinical relevance of GIT1 expression by IHC in a cohort of ER positive breast cancer samples with involved synchronous lymph nodes. We observed a significant reduction in GIT1 expression in lymph node metastasis compared to matched primary breast tumours. Although Chan *et al.* reported that GIT1 is over-expressed in lymph nodes when compared to primary breast cancer, very few cases were examined by qRT-PCR (<30) and even fewer (<10) by IHC ^6^, the most prevalent biomarker detection technique used in clinics. Furthermore, this study did not examine the potential prognostic value of GIT1 expression or its association with distant metastasis.

Our study not only included a much larger cohort of patients (n=140) measured by IHC, but we observed the same pattern of decreased *GIT1* expression in our *in silico* analysis (see below). Moreover, a reduction in GIT1 expression in metastatic tumour cells is consistent with reports of increased Arf1 and Arf6 expression in high grade tumors compared to low grade tumors in gastric, prostate and brain, as well as in breast cancer where cell lines with high invasive activities expressed higher amounts of Arf6 protein than those in weakly invasive and non-invasive cell lines ^26– 28^.

We observed that over a third of cases displayed a spatial intratumoural heterogeneous pattern of GIT1 expression between the primary tumour and the lymph node, with loss of GIT1 expression in lymph nodes being more common than its gain. Heterogeneity in protein expression is a well-established phenomenon in breast cancer, particularly regarding hormone receptor status, and has been associated with prognostic outcome ^29^. However, these studies generally report lower levels of heterogeneity (<20%) between primary and synchrononus lymph node metastases ^7, 30^, suggesting that GIT1 could be a more sensitive indicator of heterogeneity in this cancer, and hence a more powerful prognostic indicator. It should be noted that those cases with heterogeneous expression of GIT1 were not the same cases as those with heterogeneous expression of hormone receptors in this cohort. Consistent with this idea, we found that loss of GIT1 in the lymph nodes (30% of patients in this study), was indicator of poor prognosis (time to recurrence and OS) by univariate analysis and an independent indicator of prognosis by multivariate analysis. It should be noted that further analysis in independent patient cohorts within a multi-centre setting is necessary to validate these findings further.

We extended these studies to other subtypes of breast cancer by looking at cohorts from publicly available databases (n= 3452) and found that GIT1 is over-expressed in breast cancer and its expression associates inversely with ER status. Furthermore, GIT1 levels were down-regulated between primary sites and distant metastases, and that was true not only in ER+ breast cancer but also other subtypes. Despite the clear evidence shown here that GIT1 is down-regulated in both lymph node and distant metastasis in breast cancer another study reported an increase in GIT1 expression between primary tumours and lymph node metastasis ^6^. However, it should be borne in mind that the study of Chan *et al.* used a much smaller cohort of patients (n=26) and moreover the hormone receptor status of these patients was not provided. As our *in silico* analysis ( Figure 3B) suggests that GIT1 expression varies significantly with ER status, it is plausible that the subtype analysed could influence the results. Our results support the notion that, at least in ER+ breast tumours, down-regulation of GIT1 in lymph node metastases is a sign of poor prognosis.

In summary, our study has shown that the expression of GIT1 in breast cancer could serve as a useful biomarker for the management of breast cancer patients in general and for ER+/LN+ patients in particular. The mechanistic reasons behind why the loss of GIT1 in these patients is indicative of poor prognosis remains to be determined, however it is tempting to suggest that further studies could lead to better management of these patients and ultimately improve their clinical outcome.

## Data availability

The data referenced by this article are under copyright with the following copyright statement: Copyright: © 2018 Goicoechea I et al.

Data associated with the article are available under the terms of the Creative Commons Zero "No rights reserved" data waiver (CC0 1.0 Public domain dedication). 

Dataset 1: Clinical data of patient cohort. Table shows patients (numbered from 1 to 105) and their clinical features including histological subgroup, tumour size, number of affected lymph nodes, histological grade, vascular lymphatic infiltration, immunohistochemical initial status, treatment and patient follow up. 10.5256/f1000research.12393.d194222 ^31^

Dataset 2: GIT1 scoring. Table shows patients (numbered from 1 to 142) and associated primary tumour and lymph node GIT1 scoring. Categorical scores are assigned as follows according to tumour cell staining intensity; 0= negative (0%); 1=1–10%; 2= 11–50%; 3= >50%. 10.5256/f1000research.12393.d194223 ^32^

Dataset 3: Series mRNA expression matrix and clinical data information. GIT1 Expression Dataset consisting of 522 primary tumors, 3 metastatic tumors, and 22 tumor-adjacent normal samples. Data was median centered by genes. Platform: Affymetrix Human Genome U133A Array. Publicly available from https://tcga-data.nci.nih.gov/docs/publications/brca_2012/. 10.5256/f1000research.12393.d194224 ^33^

Dataset 4: Series mRNA expression matrix. Expression Dataset consisting of 2000 breast carcinoma. Platform: Affymetrix Human HT-12 V3 Array. Publicly available from http://www.cbioportal.org/study?id=brca_metabric#summary 10.5256/f1000research.12393.d194225 ^34^

Dataset 5: Series mRNA expression matrix. Expression Dataset consisting of one hundred fifty-four (154) invasive breast carcinoma samples and 4 normal breast samples. Platform: Agilent UNC Perou Lab Homo sapiens 1X44K Custom Array. Publicly available from Gluck Breast dataset ( https://www.oncomine.org) 10.5256/f1000research.12393.d194226 ^35^

Dataset 6: Series mRNA expression matrix. Expression Dataset consisting of 252 lymph-node negative breast cancer samples. Platform: Affymetrix Human Genome U133A Array. Publicly available from https://www.ncbi.nlm.nih.gov/geo/query/acc.cgi?acc=gse2034 10.5256/f1000research.12393.d194227 ^36^

Dataset 7: Series mRNA expression matrix. Expression Dataset consisting of 67 triple negative breast cancer samples. Platform: Affymetrix Human Genome U133A Array. Publicly available from https://www.ncbi.nlm.nih.gov/geo/query/acc.cgi?acc=GSE31519 10.5256/f1000research.12393.d194228 ^37^

Dataset 8: Series mRNA expression matrix. Expression Dataset consisting of 19 HER2+ brain metastasis breast cancer samples and 19 HER2+ non-metastatic breast cancer samples. Platform: Affymetrix Human X3P Array. Publicly available from https://www.ncbi.nlm.nih.gov/geo/query/acc.cgi?acc=GSE43837 10.5256/f1000research.12393.d194229 ^38^

## Consent

Written informed consent for publication of the patients' details and their images was obtained from the patients.

## Supplementary material

**Supplementary Figure S1: Examples of GIT1 expression in whole tumour sections.** Images were enhanced from the original ( Supplementary Figure S3). Patient **A** demonstrates a +/- (positive primary/negative lymph node) pattern of GIT1 expression in both TMA and whole section staining. Patient **B** shows a -/+ (weak positive primary/positive lymph node) pattern of GIT1 expression. Magnification x40.

**Supplementary Figure S2: Example of GIT1 expression patterns found in ER+ breast cancer.** Original representative intensity staining photos of GIT1 expression (primary tumour) of Figure 1. **A**. negative (score=0); **B**. weak (score=1); **C.** moderate (score=2); and **D.** strong (score=3). Magnification x400.

**Supplementary Figure S3: Examples of GIT1 expression in whole tumour sections.** Original photos of Supplementary Figure S1. Patient **A** demonstrates a +/- (positive primary/negative lymph node) pattern of GIT1 expression in both TMA and whole section staining. Patient **B** shows a -/+ (weak positive primary/positive lymph node) pattern of GIT1 expression. Magnification x40.
