# Supplementary figures and images for: Spatial intratumoural heterogeneity in the expression of GIT1 is associated with poor prognostic outcome in oestrogen receptor positive breast cancer patients with synchronous lymph node metastases
Source: F1000Res. 2018 Feb 14;6:1606. Originally published 2017 Aug 30. [Version 2] doi: 10.12688/f1000research.12393.2 (PMC5843846; doi:10.12688/f1000research.12393.2)

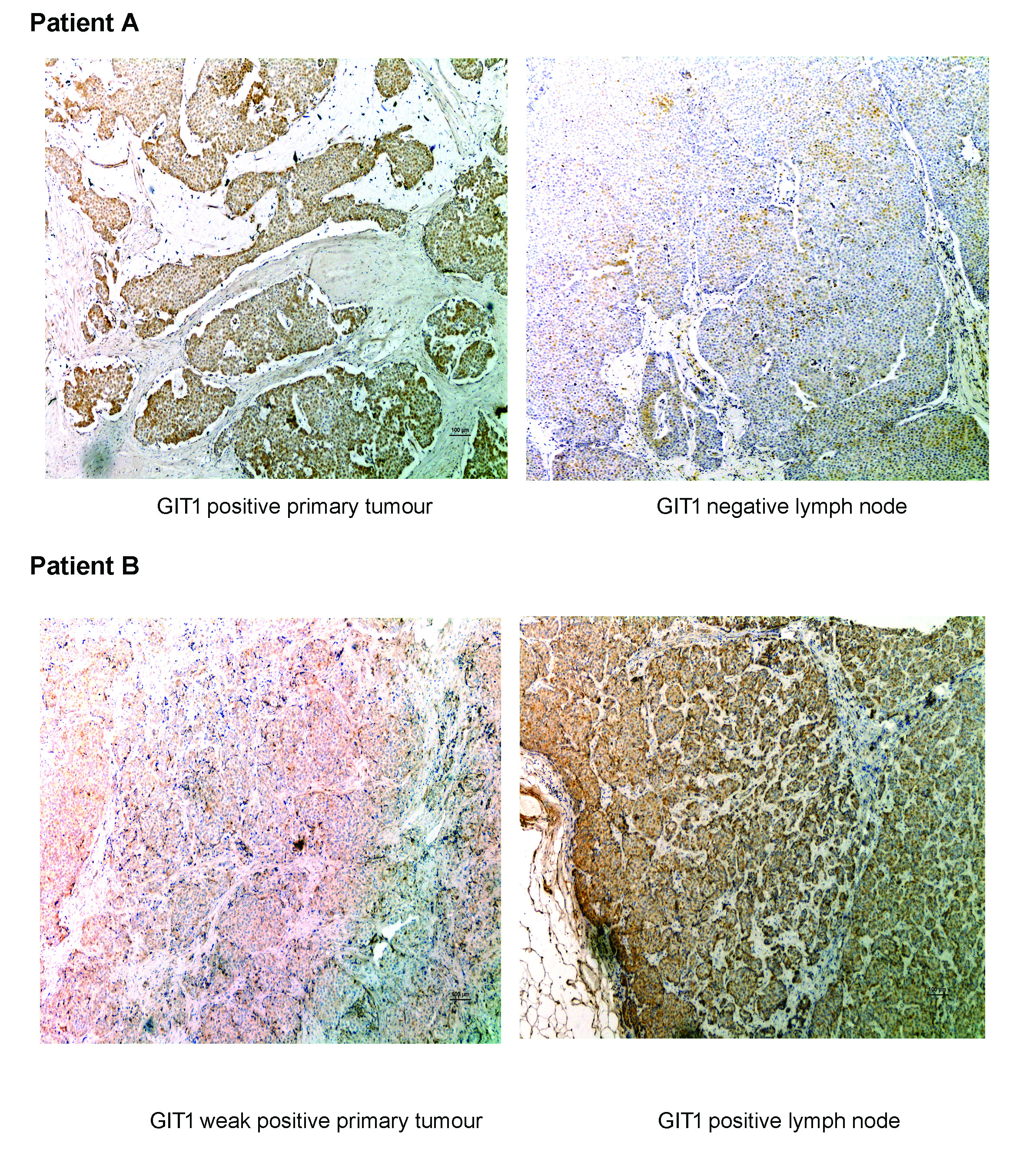

Supplement: Supplementary file 9 [file f1000research-6-14979-s0008.tgz › f2da2b29-e923-4e75-b6b0-29839ec4f875.tif]
