# Supplementary material for: Spatial intratumoural heterogeneity in the expression of GIT1 is associated with poor prognostic outcome in oestrogen receptor positive breast cancer patients with synchronous lymph node metastases
Source: F1000Res. 2018 Feb 14;6:1606. Originally published 2017 Aug 30. [Version 2] doi: 10.12688/f1000research.12393.2 (PMC5843846; doi:10.12688/f1000research.12393.2)
Supplement: Peer review history

# Peer review: Referee response for version 2

**DOI:** 10.5256/f1000research.14979.r30852

**Published:** 2018-03-07

**Authors:** Richard T. Premont

**Affiliations:** 1 Liver Center and Duke Institute for Brain Sciences, Duke University, Durham, NC, USA

The authors have addressed the major concerns raised in the original review.

I have read this submission. I believe that I have an appropriate level of expertise to confirm that it is of an acceptable scientific standard.

# Peer review: Referee response for version 2

**DOI:** 10.5256/f1000research.14979.r30851

**Published:** 2018-02-21

**Authors:** Mona M. Mohamed

**Affiliations:** 1 Department of Zoology, Cancer Biology Research Laboratory (CBRL), Faculty of Science, Cairo University, Giza, Egypt

The authors responded to all comments.

I have read this submission. I believe that I have an appropriate level of expertise to confirm that it is of an acceptable scientific standard.

# Peer review: Referee response for version 1

**DOI:** 10.5256/f1000research.13421.r27272

**Published:** 2017-11-06

**Authors:** Richard T. Premont

**Affiliations:** 1 Liver Center and Duke Institute for Brain Sciences, Duke University, Durham, NC, USA

Goicoechea et al. have examined GIT1 expression in primary breast cancers and metastatic lymph nodes a cohort of Estrogen Receptor positive (ER+) patients. While high or low GIT1 overexpression in the primary tumor does not associate with patient survival, a discordance between high GIT1 in primary tumor and low GIT1 in metastatic lymph nodes is significantly associated with reduced time to metastatic recurrence and reduced patient survival. The authors suggest that this discordance is an independent prognostic indicator of poor outcome in ER+ patients. A further analysis of published datasets shows that GIT1 mRNA level is elevated in multiple breast cancer subtypes, but this overexpression is lowest in ER+ cancers, and that low GIT1 is associated with worse outcomes in triple negative breast cancer.

 The strength of the paper is in the survival curves in Figure 2, showing that the GIT1-high primary tumor/GIT1-low lymph node metastatic (+/-) subgroup is significantly worse off than patients with either always high or always low GIT1 level. This could be a very significant finding.

 However, there are several major concerns: 

1. The study relies on the specificity of a single GIT1 polyclonal antiserum from Bethyl. The authors should list the specific lots of antisera utilized, and describe the characterization they have performed to be assured that this antiserum is in fact detecting GIT1, and not also the related GIT2 protein or even other unrelated proteins. Is similar IHC data obtained using distinct anti-GIT1 antisera? The authors should also show and point out adjacent normal breast epithelial tissue (and lymph node tissue) for comparison. The authors should consider making all primary IHC image files available as supplemental material.
2. Regarding the metastatic lymph nodes, what evidence is there that these cells are in fact metastatic? If other markers were used, they should be mentioned. The histology of the lymph nodes in Supp Fig 1, at the magnification shown, makes it impossible to distinguish normal lymphocytes from metastatic epithelial-like cells. Again, it would be helpful to include and mark adjacent non-tumor tissue.
3. The IHC figures should include scale bars in each micrograph for size. It is particularly worrying in Figure 1A that the cell nuclei are all substantially larger than those in the other panels of that figure. The cells themselves also appear larger.
4. Table 5 appears confusing because the description is incomplete. Please clarify what was compared in each case (likely just explicitly referring to Table 4). That is, among the patient cohort, what number and percentage of patients had distant metastasis? Define what histology grades are compared here (I vs II vs III, pooling of subgroups I and II vs III, etc)? What are +/- patients compared to, all other patients or just a subgroup? The table as shown also does not include a description of how the p=0.002 value reported in the text was obtained. Surely the Hazard ratio of 300000+ in Table 5 is a typo?
5. The discussion of the functions and cancer-associations of GIT1 is inadequate and incorrect. GIT1 is not “part of the Arf and Rho families of GTPases” as it is not a GTPase itself, but instead is a GTPase regulator (GTPase activating protein - deactivator - for Arf). GIT1 functional interactions with the Rho pathway, and with PAK, require an additional GIT1 partner, PIX, which is a Rho guanine nucleotide exchange factor (activator). The authors should carefully read two recent reviews that discuss GIT1 and cancer to distill the current understanding of GIT1 function ^1^^,^^2^, and then present a clearer and more accurate description of what GIT1 does and therefore why this discordance between primary and metastatic tumor might explain the differential patient survival rates they have discovered. 

 

 Minor presentation/formatting issues: 

1. The authors need to be consistent in their presentation of numerical values. Values are presented in the European style with a comma separating a number from fractional values in all of the data tables (“4,843”), but in the American style using a period in the text itself (“4.843”). This is confusing.
2. The datasets are provided as Excel spreadsheets, but with all values for each patient shown within a single cell, separated by semicolons. It would make this data more immediately accessible to provide either Excel spreadsheets with the distinct values for each patient in individual cells already, or to provide this data in a less platform-dependent manner, such as CSV tables with commas between values for each patient, which can be readily imported into any statistics program.
3. In Fig 2A and Fig 3E, GIT1 is labeled as “GIT-1”
4. It is beyond the scope of this study, but an interesting mechanistic question is, since GIT1 works hand-in-hand with PIX proteins and they regulate each others’ stability and expression, do PIX levels also change in GIT1 high vs low expressing tumors?

I have read this submission. I believe that I have an appropriate level of expertise to confirm that it is of an acceptable scientific standard, however I have significant reservations, as outlined above.

## References

[1] YooSMCerioneRAAntonyakMA: The Arf-GAP and Protein Scaffold Cat1/Git1 as a Multifaceted Regulator of Cancer Progression. *Small GTPases*.2017; 10.1080/21541248.2017.1362496 0 10.1080/21541248.2017.1362496 28981399PMC7053942

[2] ZhouWLiXPremontRT: Expanding functions of GIT Arf GTPase-activating proteins, PIX Rho guanine nucleotide exchange factors and GIT-PIX complexes. *J Cell Sci*.2016;129(10) : 10.1242/jcs.179465 1963-74 10.1242/jcs.179465 27182061PMC6518221

# Response

**Published:** 2018-02-13

**Authors:** Ibai Goicoechea

**Affiliations:** Biodonostia, Spain

Thank you very much for the useful comments made by this reviewer which have made further enriched the revised manuscript. I hope we are able to satisfy most of your concerns regarding the manuscript. In response to the specific points:

1. *The study relies on the specificity of a single GIT1 polyclonal antiserum from Bethyl. The authors should list the specific lots of antisera utilized, and describe the characterization they have performed to be assured that this antiserum is in fact detecting GIT1, and not also the related GIT2 protein or even other unrelated proteins. Is similar IHC data obtained using distinct anti-GIT1 antisera? The authors should also show and point out adjacent normal breast epithelial tissue (and lymph node tissue) for comparison. The authors should consider making all primary IHC image files available as supplemental material.*

We agree with the reviewer that many of the conclusions drawn in this study are dependent upon the specificity of the antibodies used. However, we would also like to point out that the immunohistochemistry results were also corroborated by our *in silico* analyses. As suggested by the reviewer, the specific lot of the antisera used has been included in the revised manuscript. Regarding the possibility of cross-specificity of the Ab with GIT2, even though the antisera is polyclonal, the immunogen used (i.e. between aa 375 and aa 425) is specific to GIT1 and this sequence is not present in GIT2. Furthermore, this antibody is the same that was used in the study of Chan et al (Oncogene 2014). That said we cannot rule out non-specific staining due to cross-reactivity against other proteins. This caveat has been made clear in the revised manuscript. Unfortunately, due to routine surgical protocols adjacent normal breast epithelial tissue was not available for these cases.

1. *Regarding the metastatic lymph nodes, what evidence is there that these cells are in fact metastatic? If other markers were used, they should be mentioned. The histology of the lymph nodes in Supp Fig 1, at the magnification shown, makes it impossible to distinguish normal lymphocytes from metastatic epithelial-like cells. Again, it would be helpful to include and mark adjacent non-tumor tissue.*

The identification of metastatic lymph nodes was carried out on the basis of H&E stains, by an expert breast cancer pathologist with more than 25 years’ experience.  We agree it would be very useful to include adjacent non-tumor tissue in this study however this material was not available as immunohistochemical staining was carried out on TMAs rather than whole sections and cores were selected on the basis of high-tumour content so did not contain a significant non-tumoral component.

*3. The IHC figures should include scale bars in each micrograph for size. It is particularly worrying in Figure 1A that the cell nuclei are all substantially larger than those in the other panels of that figure. The cells themselves also appear larger.*

 The figures all represent magnified fields at 400x magnification and scale bars have been added to the figure as suggested.

1. *Table 5 appears confusing because the description is incomplete. Please clarify what was compared in each case (likely just explicitly referring to Table 4). That is, among the patient cohort, what number and percentage of patients had distant metastasis?* *Define what histology grades are compared here (I vs II vs III, pooling of subgroups I and II vs III, etc)? What are +/- patients compared to, all other patients or just a subgroup? The table as shown also does not include a description of how the p=0.002 value reported in the text was obtained. Surely the Hazard ratio of 300000+ in Table 5 is a typo?*

We agree that the description of Table 5 could be confusing and have therefore changed the description accordingly.

1. *The discussion of the functions and cancer-associations of GIT1 is inadequate and incorrect. GIT1 is not “part of the Arf and Rho families of GTPases” as it is not a GTPase itself, but instead is a GTPase regulator (GTPase activating protein - deactivator - for Arf). GIT1 functional interactions with the Rho pathway, and with PAK, require an additional GIT1 partner, PIX, which is a Rho guanine nucleotide exchange factor (activator). The authors should carefully read two recent reviews that discuss GIT1 and cancer to distill the current understanding of GIT1 function* *^1^* *^,^* *^2^* *, and then present a clearer and more accurate description of what GIT1 does and therefore why this discordance between primary and metastatic tumor might explain the differential patient survival rates they have discovered. *

We thank the reviewer for pointing out these references and the information contained within. The discussion has been re-written to reflect the comments of the reviewer.

 Minor presentation/formatting issues: 

1. The authors need to be consistent in their presentation of numerical values. Values are presented in the European style with a comma separating a number from fractional values in all of the data tables (“4,843”), but in the American style using a period in the text itself (“4.843”). This is confusing.

This has been changed in the revised manuscript

1. The datasets are provided as Excel spreadsheets, but with all values for each patient shown within a single cell, separated by semicolons. It would make this data more immediately accessible to provide either Excel spreadsheets with the distinct values for each patient in individual cells already, or to provide this data in a less platform-dependent manner, such as CSV tables with commas between values for each patient, which can be readily imported into any statistics program.

This has been changed in the revised manuscript

1. In Fig 2A and Fig 3E, GIT1 is labeled as “GIT-1”
2. It is beyond the scope of this study, but an interesting mechanistic question is, since GIT1 works hand-in-hand with PIX proteins and they regulate each others’ stability and expression, do PIX levels also change in GIT1 high vs low expressing tumors?

# Peer review: Referee response for version 1

**DOI:** 10.5256/f1000research.13421.r25548

**Published:** 2017-10-05

**Authors:** Mona M. Mohamed

**Affiliations:** 1 Department of Zoology, Cancer Biology Research Laboratory (CBRL), Faculty of Science, Cairo University, Giza, Egypt

**Abstract**

 The authors mentioned that "GIT1 expression in tumour cells was scored and statistical correlation analyses were carried out". Authors should point with arrow to all tumour cells showing stain of GIT1 in tissue and differentiate between expression of GIT1 by cancer cells and stromal cells.

 "We observed that loss fo GIT1 expression in the metastasis was associated with a shorter time to recurrence, poorer overall survival, and a shorter median survival time."

 Authors should explain here that loss of "GIT1 expression" by which cells? Tumor metastatic cells in the lymph node or by cell populations of the lymph nodes (example: immune cells)?

 In material and methods scoring of GIT1 is only in tumor cells so which cells are scored in lymph nodes. Please note that the cell populations of lymph nodes (LNs) is different from that of the tumor microenvironment and authors depends on scoring of GIT1 expression by the tumor cells (less in LNs)  so this should be clarified by authors, since the IHC microscopic images provided is confusing and may show non-specific stain. 

**Methods**

 "All primary site tumours were ER+ and 91% of them PR+."

 The authors should include ER- (ER negative) patients as a control to the studied group to provide their hypothesis that **’**the expression of GIT1 in breast cancer could serve as a useful biomarker for the management of breast cancer patients in general and for ER+/LN+ patients in particular."

 Although the paper depends mainly on IHC the auhtors did not mention appropriate chromogen/substrate used to develop the color visualized using the microscope. This section should be written in details.

 I cannot read the Dataset files provided by the author, the columns and rows overlap.

**Statistical analysis**

 "Chi-square statistical test was used to determine association between GIT1 expression and lymph node metastasis (Table 3)"

 What type of correlation test used here?

 Do the authors correlate between GIT1 expression in primary tumor and the "number of metastatic lymph node" this section should be clarified?

**Results**

 The Supplementary Figures S1, S2 and S3 representing microscopic images for GIT1 show non-specific stains. The authors should present better microscopic images with high magnification showing the pattern of GIT1 by carcinoma cells and stromal cells.

 "Spatial intratumoural heterogeneity of GIT1 expression in ER+ breast cancer: When we compared GIT1 expression in primary tumours with that of their counterpart lymph node metastases surprisingly we observed a significant decrease of GIT1 expression (p=0.0054, Table 3)."

 The authors did not take in consideration that cellular population of LN is different from that of carcinoma cells. A better IHC images should be presented showing which cell population express GIT1 in each of carcinoma tissues and lymph nodes.

 "A comparison of GIT1 expression between ER+ and ER- tumours in two independent Datasets demonstrated a significantly lower level of expression in ER+ tumours compared to ER- tumours (8,11)."

 The references (8,11); assess GIT1 at mRNA level not at the protein level, in the present study authors should include ER- patients and their associated lymph nodes as control group to ER+.

**Discussion**

 Should be re-written presenting the mechanisms of GIT1 in carcinogenesis and scientific explanation for the authors findings "loss of GIT1 expression in the metastasis was associated with a shorter time to recurrence, poorer overall survival, and a shorter median survival time".

I have read this submission. I believe that I have an appropriate level of expertise to confirm that it is of an acceptable scientific standard, however I have significant reservations, as outlined above.

# Response

**Published:** 2018-01-17

**Authors:** Ibai Goicoechea

**Affiliations:** Biodonostia, Spain

We thank the reviewer for their insightful comments and have made changes in the revised manuscript to address these concerns. Specifically:

**Abstract**

*P1: The authors mentioned that "GIT1 expression in tumour cells was scored and statistical correlation analyses were carried out". Authors should point with arrow to all tumour cells showing stain of GIT1 in tissue and differentiate between expression of GIT1 by cancer cells and stromal cells.*

**

***Only tumor cells were scored in this analysis not stromal or other microenvironment cell types***

*"We observed that loss fo GIT1 expression in the metastasis was associated with a shorter time to recurrence, poorer overall survival, and a shorter median survival time."*

*Authors should explain here that loss of "GIT1 expression" by which cells? Tumor metastatic cells in the lymph node or by cell populations of the lymph nodes (example: immune cells)?*

**

***Tumor cells (text changed in revised manuscript)***

*In material and methods scoring of GIT1 is only in tumor cells so which cells are scored in lymph nodes. Please note that the cell populations of lymph nodes (LNs) is different from that of the tumor microenvironment and authors depends on scoring of GIT1 expression by the tumor cells (less in LNs)  so this should be clarified by authors, since the IHC microscopic images provided is confusing and may show non-specific stain.*

**Only tumor cells were scored in the involved lymph nodes, non-involved lymph nodes were used as negative controls in this instance.**

**Methods**

*"All primary site tumours were ER+ and 91% of them PR+."*

*The authors should include ER- (ER negative) patients as a control to the studied group to provide their hypothesis that **’**the expression of GIT1 in breast cancer could serve as a useful biomarker for the management of breast cancer patients in general and for ER+/LN+ patients in particular."*

**

***We agree that this would have been a good comparison to carry out. Unfortunately there were insufficient suitable ER- cases available for testing. We also decided to focus the study on ER+ cases as representing the majority of breast cancer cases***

*Although the paper depends mainly on IHC the auhtors did not mention appropriate chromogen/substrate used to develop the color visualized using the microscope. This section should be written in details.*

**The chromagen used was DAB. This information has been included in the revised manuscript**

*I cannot read the Dataset files provided by the author, the columns and rows overlap.*

**

***I have informed the editor of this problem***

**Statistical analysis**

*"Chi-square statistical test was used to determine association between GIT1 expression and lymph node metastasis (Table 3)"*

*What type of correlation test used here?*

**

***This was Chi-square analysis***

*Do the authors correlate between GIT1 expression in primary tumor and the "number of metastatic lymph node" this section should be clarified?*

***This is an interesting point and we have added this analysis to the revised manuscript. There was no correlation.***

**Results**

*The Supplementary Figures S1, S2 and S3 representing microscopic images for GIT1 show non-specific stains. The authors should present better microscopic images with high magnification showing the pattern of GIT1 by carcinoma cells and stromal cells.*

**

***Whilst we agree with the reviewer that some GIT1 expression was non-specific this was very different staining from that seen in tumor tissue. This can be seen clearly from the figures S1  and a high magnification image is already given in Figure 1 whereby some light stromal staining is seen in the panel A compared to string tumour staining in panel D.***

*"Spatial intratumoural heterogeneity of GIT1 expression in ER+ breast cancer: When we compared GIT1 expression in primary tumours with that of their counterpart lymph node metastases surprisingly we observed a significant decrease of GIT1 expression (p=0.0054, Table 3)."*

**

*The authors did not take in consideration that cellular population of LN is different from that of carcinoma cells. A better IHC images should be presented showing which cell population express GIT1 in each of carcinoma tissues and lymph nodes.*

**We disagree with the reviewer that we did not take into consideration the difference in non-tumoral staining between lymph node and primary material. The scoring was carried out by a breast pathologist with more than 25 years experience in the field. These differences are clearly shown in Supplementary Figure S1 which shows representative images of negative and positive lymph node staining, as well as primary tumor material.**

*"A comparison of GIT1 expression between ER+ and ER- tumours in two independent Datasets demonstrated a significantly lower level of expression in ER+ tumours compared to ER- tumours (8,11)."*

**

*The references (8,11); assess GIT1 at mRNA level not at the protein level, in the present study authors should include ER- patients and their associated lymph nodes as control group to ER+.*

**

***As stated above we would have liked to included ER- cases as well. However, that was not possible***

**Discussion**

*Should be re-written presenting the mechanisms of GIT1 in carcinogenesis and scientific explanation for the authors findings "loss of GIT1 expression in the metastasis was associated with a shorter time to recurrence, poorer overall survival, and a shorter median survival tim*e".

**The discussion has been rewritten to add possible explanations for this statement**
